# Supplementary material for: Chitin-based barrier immunity and its loss predated mucus-colonization by indigenous gut microbiota
Source: Nat Commun. 2018 Aug 24;9:3402. doi: 10.1038/s41467-018-05884-0 (PMC6109156; doi:10.1038/s41467-018-05884-0)
Supplement: Supplementary file 1 — Supplementary Information [file 41467_2018_5884_MOESM1_ESM.pdf]

Supplementary Information

Chitin-based barrier immunity and its loss predated the mucus-colonization by indigenous gut microbiota

Nakashima *et al*.

Supplementary Figures 1-6.

Supplementary Tables 1-3.

Supplementary References.

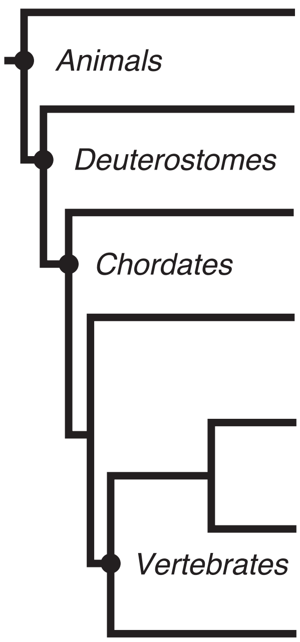

	Gill slits	Endostyle	Pulley cartilage	Jaw
Others	No	No	No	No
Hemichordates /Echinoderms	Yes*	No	No	No
Lancelets	Yes	Yes	No	No
Tunicates	Yes	Yes	No	No
Lampreys	Yes	Yes**	Yes	No
Hagfish	Yes	No	Yes	No
Jawed vertebrates	Yes	No	No	Yes

Supplementary Figure 1. Diversification of food habit in chordates accompanied morphological innovations in the pharyngeal region. The left diagram shows relationships of chordates in animal phylogeny. Chordates belong to deuterostomes. Non-deuterostome animals (*e.g.* protostomes, cnidarians and sponges) are collectively shown as others. The right panel shows the presence or absence of four pharyngeal anatomical features in each group. The most prominent and oldest food habit in marine environments is suspension-feeding, which captures particulate organic matter in seawater using various mechanisms particular to each animal group[1]. Gill slits are a deuterostome innovation that enables internal water flow through the pharynx[2]. Internal water flow, produced by cilia surrounding gill slits, is functionally important for mucociliary deposit-feeding in enteropneust hemichordates[3]. Gill slits were lost secondarily in pterobranch hemichordates and echinoderms that show diverse food habits*. The endostyle, or the longitudinal glandular groove on the ventral wall of the pharynx, is a chordate innovation that secretes unique mucus nets for high-efficiency filtration of internal water flow[4,5]. This internal filter-feeding using mucus nets occurs in two invertebrate chordates, lancelets and tunicates (highlighted in yellow). In the jawless vertebrate lampreys, the endostyle persists only in larval forms** and no longer secretes mucus nets. The lamprey endostyle transforms during metamorphosis to the follicular thyroid of adult forms. Concomitantly, larval suspension-feeding using mucus cords, which are produced by goblet cells, shifts to adult blood/flesh-feeding[6]. The other jawless vertebrates, hagfish, which lack a larval stage, are predators that prey upon various invertebrates, but are opportunistic scavengers on animal remains[7]. Despite different food habits, adult lampreys and hagfish share a common feeding mechanism using pulley movements of lingual cartilage, a vertebrate innovation[8,9] (highlighted in red). In jawed vertebrates, subsequent morphological innovations in the pharynx (*e.g.* jaw, tongue and dentition), together with those in the digestive tract (*e.g.* acidic stomach, rumen and cecum) are relevant to diversification of food habits including herbivory[10] (highlighted in blue).

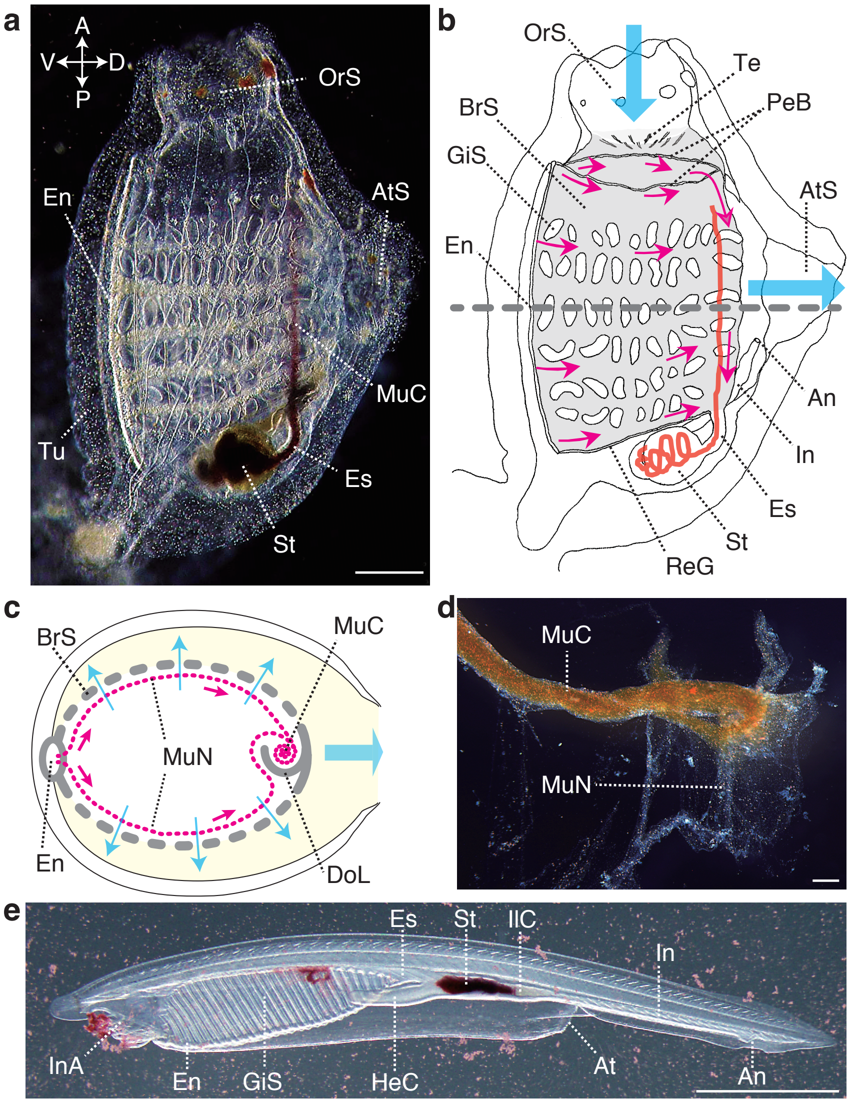

Supplementary Figure 2. Internal filter-feeding using mucus nets of invertebrate chordates. **(a-c)** The feeding mechanism of the tunicate *Ciona intestinalis* Type A (**a**, body image; **b**, anatomy; **c**, horizontal section at the dotted line in **b**). A cross indicates the body axes: A, anterior; P, posterior; D, dorsal; V, ventral. The largest organ of the body is the branchial sac (BrS, gray shaded in **b**), an enlarged pharynx perforated with ciliated gill slits (GiS). Coordinated movements of these cilia generate mechanical force to draw seawater from the oral siphon (OrS) into the branchial sac (a vertical cyan arrow in **b**). Seawater passes through the gill slits (radial cyan arrows in **c**) to enter the atrial space (highlighted in yellow in **c**) and is expelled from the atrial siphon (AtS) (horizontal cyan arrows in **b** and **c**). Particulate matter in seawater is trapped with two sheets of mucus net (MuN) that cover the left and right branchial walls (magenta dotted lines in **c**). Mucus nets are secreted from the glandular endostyle (En) on the ventral branchial wall[11] and conveyed dorsally by ciliary actions of the peripharyngeal bands (PeB), the retropharyngeal groove (ReG), and the internal surface of the branchial sac (horizontal magenta arrows in **b**). At the dorsal lamina (DoL), mucus nets are rolled up as a single mucus cord (MuC), which is then transported posteriorly to the esophagus (Es) and the stomach (St), where the cord often coils until it enters the intestine (In) (vertical magenta arrows in **b**). The mucus cord is recognizable due to trapped red beads. The intestine turns anterior-dorsally, and the anus (An) opens near the atrial siphon, from which feces are expelled into the excurrent water. For feeding operations, see Supplementary Movie 1. Te, tentacles; Tu, tunic. **(d)** A terminus of mucus cord (MuC) partially unfolded to mucus nets (MuN) with trapped red beads. **(e)** The lancelet *Branchiostoma floridae* is an internal filter-feeder using mucus nets[12]. For operations, see Supplementary Movie 2. InA, inhalant aperture; HeC, hepatic cecum; IlC, ilio-colon; At, atripore. Scale bars (**a,d**), 100 μm and (**e**), 200 μm.

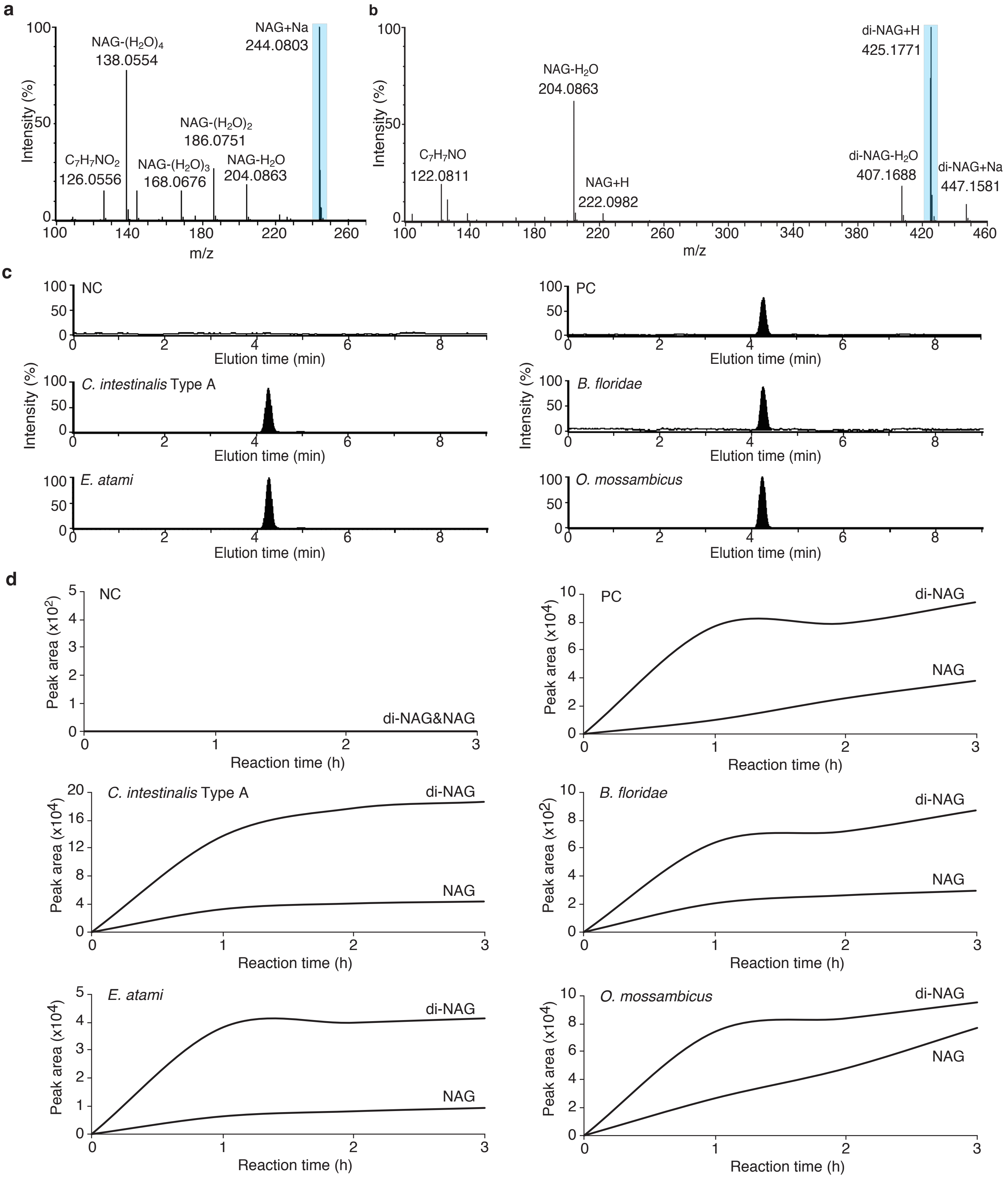

Supplementary Figure 3. Mass spectrometric analysis of chitinase products of intestinal barrier membranes. (a-b) LC-MS spectra of N-acetylglucosamine (NAG) and N-acetylchitobiose (di-NAG) standards. NAG+Na (m/z 244.0083) and di-NAG+H (m/z 425.1771) are selected for subsequent quantification (highlighted in blue). **(c)** Extracted ion chromatograms for NAG released from barrier membrane samples by *Pyrococcus furiosus* chitinase. This hyperthermophilic chitinase releases NAG and di-NAG from chitin as the main product[13]. Species names indicate the origin of samples. NC, negative control lacking chitinase in reaction; PC, positive control (chitin standard). **(d)** Time course relative quantification of released NAG and di-NAG. Peak area of extracted ion chromatograms is plotted.

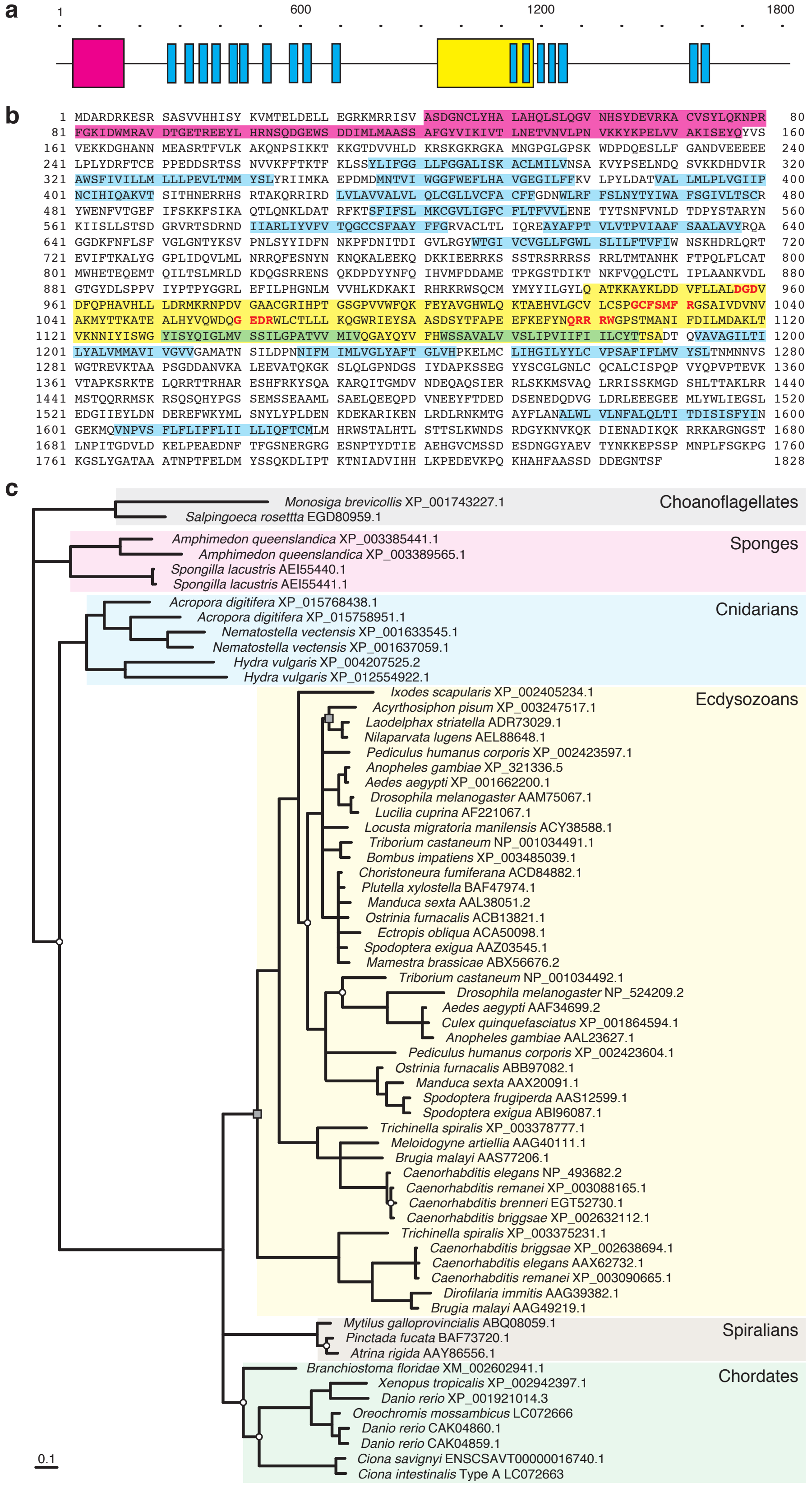

Supplementary Figure 4. ***Ciona* chitin synthase (Ci-CHS). (a)** Domain structure of Ci-CHS. Ci-CHS contains an N-terminal ovarian tumor domain (a magenta box), 17 transmembrane domains (cyan boxes) and a chitin synthase domain (a yellow box). **(b)** Amino acid sequence of Ci-CHS (1,828 amino acid residues). Numerals on the left and right sides of the sequence denote the number of amino acids from the putative translation-initiating methionine. Regions corresponding to the domains are shown by coloring as in **a**. Motif sequences of chitin synthases are typed in red. **(c)** Bayesian inference topology of animal chitin synthases. Species names are followed by accession codes to Genbank or Ensambl. Choanoflagellate sequences are used as outgroups. Plain nodes, gray rectangles and white circles denote posterior probabilities of 1.0, >0.95 or >0.9, respectively.

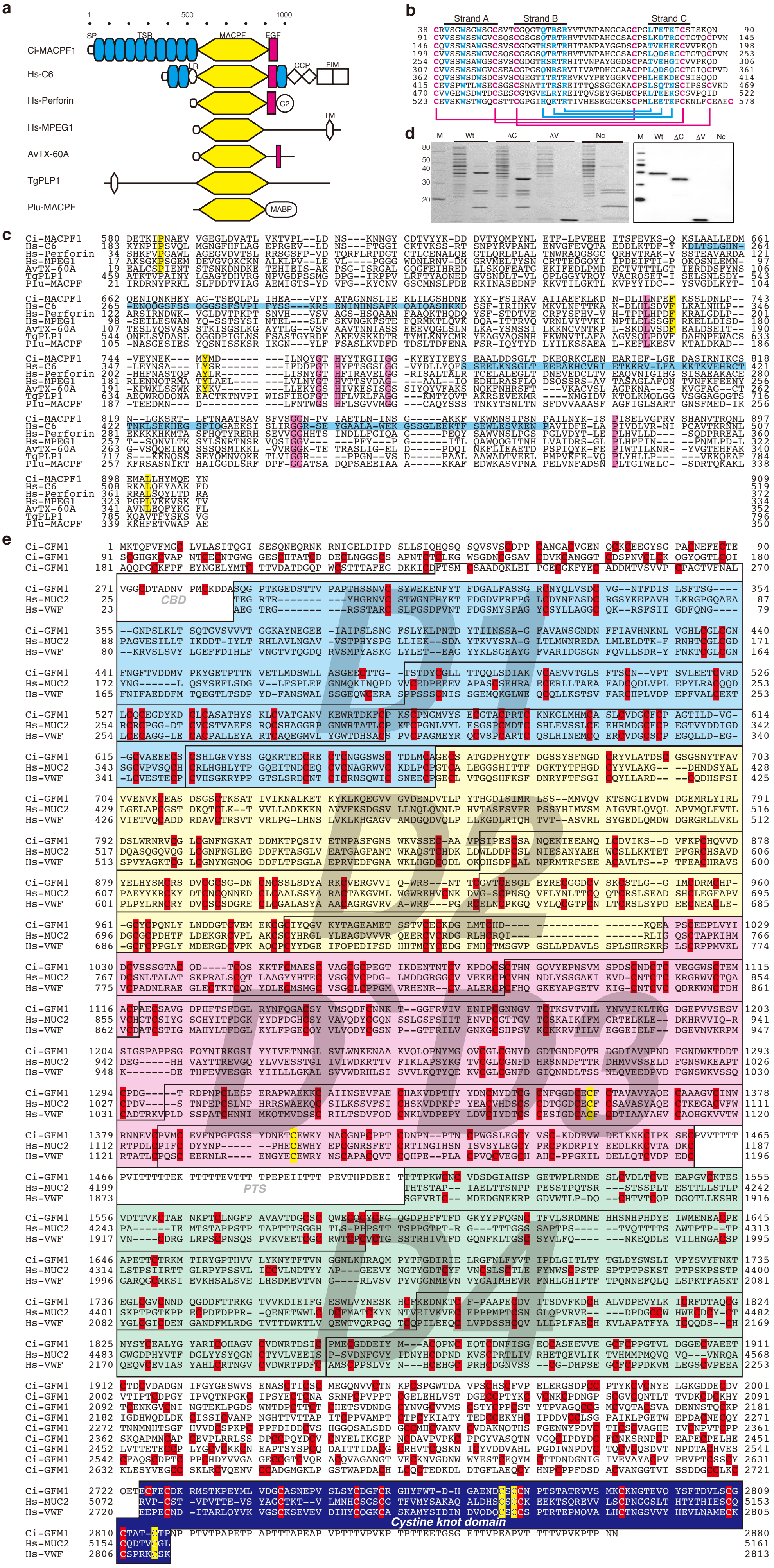

a
Ci-MACPF1
Hs-C6
Hs-Perforin
Hs-MPEG1
AvTX-60A
TgPLP1
Plu-MACPF
SP
TSR
LR
MACPF
EGF
CCP
FIM
C2
TM
MABP
b
Strand A
Strand B
Strand C
d
M
Wt
ΔC
ΔV
Nc
c
e
Ci-GFM1
Hs-MUC2
Hs-VWF
CBD
PTS
Cystine knot domain

Supplementary Figure 5. Protein components of *Ciona* barrier membranes. (**a**) Domain structure of Ci-MACPF1. MACPF domains (yellow) are essential for cytolytic activities in different biological contexts[14]. Hs-C6, human complement factor 6; Hs-Perforin, human perforin; Hs-MPEG1, the common ancestor of C6 and perforin; AvTX-60A, a nematocyst toxin of the sea anemone *Actineria villosa*; TgPLP1, an egression and virulence factor of the apicomplexan malaria pathogen *Toxoplasma gondii* and Plu-MACPF, a bacterial MACPF of *Photorhabdus luminescens*. SP, signal peptide; TSR, thrombospondin type 1 repeats (cyan); EGF, epidermal growth factor-like domain (magenta); CCP, complement control protein domain; FIM, factor I/membrane attack complex domain; C2, Ca^{2+} dependent membrane-targeting domain and MABP, multi-vesicular body 12-associated beta-prism domain. (**b**) Alignment of 10 TSRs of Ci-MACPF1. TSRs form three-stranded folds stabilized by inter-strand interactions[15]. Lines below sequences denote predicted interactions between conserved residues (cyan) and cysteines (magenta). The predicted disulfide bonds follow the pattern of TSR subgroup 2, which binds to extracellular glycosaminoglycans. (**c**) Alignment of MACPF domains. Sequence ID follows **a**. Conserved residues for all or animal sequences are highlighted in red or yellow, respectively. Despite this low level of similarity, a common folding pattern and conformational changes underlying cytolytic functions are conserved between the human and bacterial sequences[14]. Blue regions correspond to clusters of helices in Hs-C6 that insert into the cellular lipid bilayer. (**d**) Heterologous expression of VCBP-C in *E. coli* (left panel, silver stain; right panel, Western blot). Wt, ΔC, ΔV and Nc (negative control) consist of two lanes: left, cell lysate and right, purified protein. M, marker. **(e)** Conservation of cysteine residues in Ci-GFM1, human MUC2 (Hs-MUC2) and human VWF (Hs-VWF). Alignments are made for D1 (cyan), D2 (yellow), D\`D3 (magenta), D4 (green) and CK (blue) with corresponding regions of Hs-MUC2 and Hs-VWF. Domains in each assembly are boxed. Cysteines are highlighted in red. Of the 157 cysteines of Ci-GFM1 in the aligned regions, 151 are conserved with at least one of the human sequences. The 5 cysteines that form intermolecular disulfide bonds in Hs-MUC2 and Hs-VWF are conserved in Ci-GFM1 (highlighted in yellow).

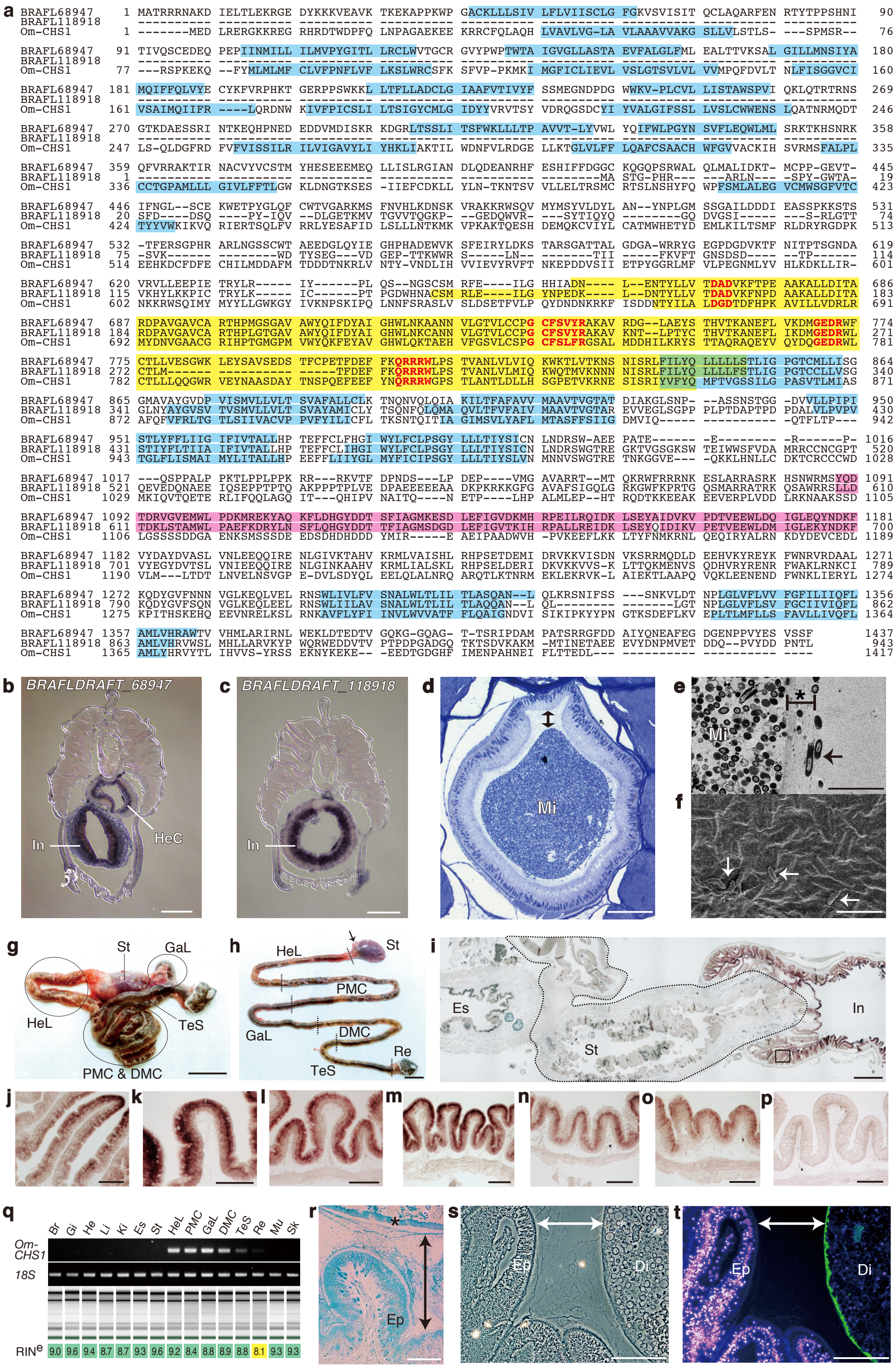
a
BRAFL68947 1 MATRRRNAKD IELTLEKRGE DYKKKVEAVK TKEKAPPKWP GACKLLLSIV LFLVIISCLG FGKVSVISIT QCLAQARFEN RTYTPPSHNI 90
BRAFL118918 ---------- ---------- ---------- ---------- ---------- ---------- ---------- ---------- ----------
Om-CHS1 1 -------MED LRERGKKREG RHRDTWDPFQ LNPAGAEKEE KRRCFQLAQH LVAVLVG-LA VLAAAVVAKG SLLVLSTLS- ---SPMSR-- 76
BRAFL68947 91 TIVQSCEDEQ PEPIINMILL ILMVPYGITL LRCLWVTGCR GVYPWPTWTA IGVGLLASTA EVFALGLFML EALTTVKSAL GILLMNSIYA 180
BRAFL118918 ---------- ---------- ---------- ---------- ---------- ---------- ---------- ---------- ----------
Om-CHS1 77 ---RSPKEKQ --FYMLMLMF CLVFPNFLVF LKSLWRCSFK SFVP-PKMKI MGFICLIEVL VSLGTSVLVL VVMPQFDVLT NLFISGGVCI 160
BRAFL68947 181 MQIFFQLVYE CYKFVRPHKT GERPPSWKKL LTFLLADCLG IAAFVTIVYF SSMEGNDPDG WWKV-PLCVL LISTAWSPVI QKLQTRTRNS 269
BRAFL118918 ---------- ---------- ---------- ---------- ---------- ---------- ---------- ---------- ----------
Om-CHS1 161 VSAIMQIIFR ----LQRDNW KIVFPICSLI LTSIGYCMLG IDYYVRVTSY VDRQGSDCYI YVALGIFSSL LVSLCWWENS LQATNRMQDT 246
BRAFL68947 270 GTKDAESSRI NTKEQHPNED EDDVMDISKR KDGRLTSSLI TSFWKLLLTP AVVT-LYVWL YQIFWLPGYN SVFLEQWLML SRKTKHSNRK 358
BRAFL118918 ---------- ---------- ---------- ---------- ---------- ---------- ---------- ---------- ----------
Om-CHS1 247 LS-QLDGFRD FVFVISSILR ILVIGAVYLI YHKLIAKTIL WDNFVLRDGE LLKTGLVLFF LQAFCSAACH WFGVVACKIH SVRMSFALPL 335
BRAFL68947 359 QFVRRAKTIR NACVYVCSTM YHESEEEMEQ LLISLRGIAN DLQDEANRHF ESHIFFDGGC KQGQPSRWAL QLMALIDKT- MCPP-GEVT- 445
BRAFL118918 1 ---------- ---------- ---------- ---------- ---------- --------MA STG-PHR--- ---ARLN--- -SPY-GWTA- 19
Om-CHS1 336 CCTGPAMLLL GIVLFFTLGW KLDNGTKSES -IIEFCDKLL YLN-TKNTSV VLLELTRSMC RTSLNSHYFQ WPFSMLALEG VCMWSGFVTC 423
BRAFL68947 446 IFNGL--SCE KWETPYGLQF CWTVGARKMS FNVHLKDNSK VRAKKRWSQV MYMSYVLDYL AN--YNPLGM SSGAILDDDI EASSPKKSTS 531
BRAFL118918 20 SFD----DSQ ----PY-IQV --DLGETKMV TGVVTQGKP- --GEDQWVR- ---SYTIQYQ --------GQ DVGSI----- ---S-RLGTT 74
Om-CHS1 424 TYYVWKIKVQ RIERTSQLFV RRLYESAFID LSLLLNTKMK VPKAKTQESH DEMQKCVIYL CATMWHETYD EMLKILTSMF RLDRYRGDPK 513
BRAFL68947 532 -TFERSGPHR ARLNGSSCWT AEEDGLQYIE GHPHADEWVK SFEIRYLDKS TARSGATTAL GDGA-WRRYG EGPDGDVKTF NITPTSGNDA 619
BRAFL118918 75 -SVK------ --------WD TYSEG---VD GEP-TTKWWR S-----LN-- -------TII -----FKVFT GNSDSD---- --TP------ 114
Om-CHS1 514 EEHKDCFDFE CHILMDDAFM TDDDTNKRLV NTY-VNDLIH VVIEVYRVFT NKEPDDVSII ETPYGGRLMF VLPEGNMLYV HLKDKLLIR- 601
BRAFL68947 620 VRVLLEEPIE TRYLR----- IY------PL QS--NGSCSM RFE----ILG HHIADN---- L--ENTYLLV TDADVKFTPE AAKALLDITA 686
BRAFL118918 115 VKHYLKKPIC TRYLK----- IC------PT PGDWHNACSM RLE----ILG YNPEDK---- L--DNTYLLV TDADVKFNPD AAKALLDITA 183
Om-CHS1 602 NKKRWSQIMY MYYLLGWKGY IVKNPSKIPQ LNNFSRASLV SLDSETFVLP QYDNDNKRKF ISDDNTYILA LDGDTDFHPK AVILLVDRLR 691
BRAFL68947 687 RDPAVGAVCA RTHPMGSGAV AWYQIFDYAI GHWLNKAANN VLGTVLCCPG CFSVYRAKAV RDG--LAEYS THVTKANEFL VKDMGEDRWF 774
BRAFL118918 184 RDPAVGAVCA RTHPLGTGAV AWYQIFDYAI GHWLNKCANN VLGTVLCCPG CFSVYRAKAV KNT--LPTYC THVTKANEFL IKDMGEDRWL 271
Om-CHS1 692 MYDNVGAACG RIHPTGMGPM VWYQKFEYAV GHWLQKTAEH VFGSVLCSPG CFSLFRGSAL MDDHILKRYS TTAQRAQEYV QYDQGEDRWL 781
BRAFL68947 775 CTLLVESGWK LEYSAVSEDS TFCPETFDEF FKQRRRWLPS TVANLVLVIQ KWKTLVTKNS NISRLFILYQ LLLLLSTLIG PGTCMLLISG 864
BRAFL118918 272 CTLM------ ---------- -----TFDEF FKQRRRWLPS TVANLVLMIQ KWQTMVKNNN NISRLFILYQ LLLLFSTLIG PGTCCLLVSG 340
Om-CHS1 782 CTLLLQQGWR VEYNAASDAY TNSPQEFEEF YNQRRRWGPS TLANTLDLLH SGPETVKRNE SISRIYVFYQ MFTVGSSILG PASVTLMIAS 871
BRAFL68947 865 GMAVAYGVDP VISMVLLVLT SVAFALLCLK TNQNVQLQIA KILTFAFAVV MAAVTVGTAT DIAKGLSNP- --ASSNSTGG -DVVLLPIPI 950
BRAFL118918 341 GLNYAYGVSV TVSMVLLVLT SVAYAMICLY TSQNFQLQMA QVLTFVFAIV MAAVTVGTAR EVVEGLSGPP PLPTDAPTPD PDALVLPVPV 430
Om-CHS1 872 AFQFVFRLTG TLSIIVACVP PVFYILICFL TKSNTQITIA GIMSVLYAFL MTASFFSIIG DMVIQ----- ---------- -QTFLTP--- 942
BRAFL68947 951 STLYFFLIIG IFIVTALLHP TEFFCLFHGI WYLFCLPSGY LLLTIYSICN LNDRSW-AEE PATE------ -E-------- -R------P- 1016
BRAFL118918 431 STIYFLTIIA IFIVTALLHP TEFFCLIHGI WYLFCLPSGY LLLTIYSICN LNDRSWGTRE GKTVGSGKSW TEIWWSFVDA MRRCCNCGPT 520
Om-CHS1 943 TGLFLISMAI MYLITALLHP EEFFLIIYGL MYFICIPSGY LLLTIYSLVN MNNVSWGTRE TNKGGVE--- -QKKLHNLLC DKTCRCCCWD 1028
BRAFL68947 1017 ---QSPPALP PKTLPPLPPK RR---RKVTF DPNDS---LP DEP----VMG AVARRT--MT QKRWFRRRNK ESLARRASRK HSNWRMSYQD 1091
BRAFL118918 521 QEVEDQNAEE IQSEPPTPTQ AKPPPTPLVE DPAEEEEEAA DKPKRKKGFG AVAFSIGQLG RKGWFKPRTG QSMARRATRK QSAWRRSLLD 610
Om-CHS1 1029 MKIQVTQETE RLIFQQLAGQ IT---QHIPV NAITQ----N ETP----LHP ALMLEP--HT RQDTKKEEAK EEVERPLVDD LRKNAAKSSD 1105
BRAFL68947 1092 TDRVGVEMWL PDKMREKYAQ KFLDHGYDDT SFIAGMKESD LEFIGVDKMH RPEILRQIDK LSEYAIDVKV PDTVEEWLDQ IGLEQYNDKF 1181
BRAFL118918 611 TDKLSTAMWL PAEFKDRYLN SFLQHGYDDT TFIAGMSDGD LEFIGVTKIH RPALLREIDK LSEYQIDIKV PETVEEWLDM IGLEKYNDKF 700
Om-CHS1 1106 LGSSSSDDGA ENKSMSSSDE EDSDHDHDDD YMIR-----E AEIPAADWVH -PVKEEFLKK LTYFNMKRNL QEQIRYALRN KDYDEVCEDL 1189
BRAFL68947 1182 VYDAYDVASL VNLEEQQIRE NLGIVKTAHV KRMLVAISHL RHPSETDEMI DRVKKVISDN VKSRRMQDLD EEHVKYREYK FWNRVRDAAL 1271
BRAFL118918 701 VYEGYDVTSL VNIEEQQIRE NLGINKAAHV KRMLIALSKL RHPSELDERI DKVKKVVS-K LTTQKMENVS QDHVRYRENR FWAKLRNKCI 789
Om-CHS1 1190 VLM---LTDT LNVELNSVGP E-DVLSDYQL EELQLALNRQ ARQTLKTNRM EKLEKRVK-L AIEKTLAAPQ VQKLEENEND FWNKLIERYL 1274
BRAFL68947 1272 KQDYGVFNNN VGLKEQLVEL RNSWLIVLFV SNALWLTLIL TLASQAN--L QLKRSNIFSS ---SNKVLDT NPLGLVFLVV FGFILIIQFL 1356
BRAFL118918 790 KQDYGVFSQN VGLKEQLEEL RNSWLIILAV SNALWLTLIL TLAQQAN--L QL-------- -------LGT NPLGLVFLSV FGCIIVIQFL 862
Om-CHS1 1275 KPITHSKEHQ EEVNRELKSL RNKAVFLYFI INVLWVVATF FLQAIGNDVI SIKIPKYYPN GTKSDEFLKV EPLTLMFLLS FAVLLIVQFL 1364
BRAFL68947 1357 AMLVHRAWTV VHMLARIRNL WEKLDTEDTV GQKG-GQAG- T-TSRIPDAM PATSRRGFDD AIYQNEAFEG DGENPPVYES VSSF 1437
BRAFL118918 863 AMLVHRVWSL MHLLARVKYP WQRWEDDVTV PTPDPGADGQ TKTSDVKAKM -MTINETAEE EVYDNPMVET DDQ--PVYDD PNTL 943
Om-CHS1 1365 AMLYHRVYTL IHVVS-YRSS EKNYKEKE-- -EEDTGDGHF IMENPAHNEI FLTTEDL--- ---------- ---------- ---- 1417
b
BRAFLDRAFT_68947
In
HeC
c
BRAFLDRAFT_118918
In
d
Mi
e
*
Mi
f
g
St
GaL
HeL
TeS
PMC & DMC
h
HeL
St
PMC
GaL
DMC
Re
TeS
i
Es
St
In
j
k
l
m
n
o
p
q
Br
Gi
He
Li
Ki
Es
St
HeL
PMC
GaL
DMC
TeS
Re
Mu
Sk
Om-CHS1
18S
RINe
9.0 9.6 9.4 8.7 8.7 9.3 9.6 9.2 8.4 8.8 8.9 8.8 8.1 9.3 9.3
r
*
Ep
s
Ep
Di
t
Ep
Di

Supplementary Figure 6. Intestinal chitin synthesis in the lancelet, *B. floridae*, and the ray-finned fish, *O. mossambicus*. (a) Alignments of putative chitin synthases of *B. floridae*, BRAFLDRAFT_68947 and BRAFLDRAFT_118918, and of *O. mossambicus*, Om-CHS1. Cyan, yellow and magenta denotes transmembrane, chitin synthase and sterile_alpha_motif domains, respectively. Motif sequences of chitin synthases are typed in red. (**b-c**) *In situ* hybridized cross-sections of *B. floridae* showing chitin synthase expression in the hepatic cecum (HeC) and the intestine (In). (**d**) Toluidine blue-stained cross-section of *B. floridae* showing an axenic space (double-headed arrow) between the gut epithelium and the barrier membrane that encloses ingested microbes (Mi). Bacterial breaches are observed in the right half of the axenic space. (**e**) A TEM image of bacterial breach. An arrow indicates microbes outside a barrier membrane (*). (**f**) An SEM image showing microbial attachments to the outer surface of the barrier membrane (arrows). (**g-h**) Gastrointestinal tract of *O. mossambicus* (**g**, coiled form; **h**, extended form). St, stomach; HeL, hepatic loop; PMC, proximal major coil; GaL, gastric loop; DMC, distal major coil; TeS, terminal segment; Re, rectum. An arrow denotes the cardia. (**i**) *In situ* hybridized sagittal section showing expression of *Om-CHS1* in the epithelium of intestine (In). Stomach is demarcated with dots. Es, esophagus. (**j**) Enlargement of the boxed area in **i**. (**k-p**) *In situ* hybridized cross-sections (**k**, HeL; **l**, PMC; **m**, GaL; **n**, DMC; **o**, TeS; **p**, PMC with sense probe). (**q**) RT-PCR of *Om-CHS1*. This composite figure shows the results of RT-PCR for *Om-CHS1* and *18S rRNA*, together with gel separation profiles and RIN^e values of RNA samples. Br, brain; Gi, gill; He, heart; Li, liver; Ki, kidney; Mu, muscle; Sk, skin. (**r**) Alcian blue-stained cross-section of PMC. The villus epithelium (Ep) is covered with a mucus layer (double-headed arrow), as in DMC. Asterisk denotes a barrier membrane. (**s-t**) CBD&DAPI-stained cross-sections of PMC (**s**, Phase-contrast; **t**, fluorescent). A chitinous membrane (green) separates digesta microbes (blue) from the mucus layer (double-headed arrows), as in DMC. Di, digesta. Scale bars (**b-d,j-p,r-t**), 100 μm; (**e,f**), 5 μm; (**g,h**), 1 cm; and (**i**), 1 mm.

Representative transcript ID	Product	Number of chitin-binding domain	Normalized protein score	Number of detected peptide
KH.C1.45.v1.A.ND1-1	Ci-MACPF1, membrane attack complex/perforin protein		221,999	2,967
KH.C4.625.v1.A.ND1-1	VCBP-C, variable-region containing chitin-binding protein	1	165,000	2,297
KH.L108.38.v2.A.ND1-1	Ci-GFM1, gel-forming mucin	1	161,356	2,095
KH.L119.18.v1.A.ND1-1	Ci-GH18, chitinase	2	76,140	1,221
KH.L141.55.v1.A.SL1-1	Angiotensin-converting enzyme, metalloprotease		68,177	947
KH.C12.673.v1.A.ND1-1	Annexin		65,741	893
KH.C8.470.v3.A.ND1-1	Serine protease inhibitor		50,104	753
KH.C9.782.v1.A.ND1-1	Ci-CLCA1, Ca-activated chloride channel	2	47,178	686
KH.S425.9.v1.A.ND1-1	Chymotrypsinogen B		42,512	537
KH.L9.11.v1.C.SL1-1	Catalase		30,305	368
KH.C4.259.v1.A.ND1-1	HEXB beta-hexosaminidase subunit beta		27,304	376
KH.L157.5.v3.A.SL1-1	Sodium/potassium-transporting ATPase subunit alpha-3		26,400	341
KH.C4.761.v1.A.ND1-1	Alcohol dehydrogenase		25,575	364
KH.C8.123.v2.A.SL2-1	Metalloprotease		24,227	304
KH.C11.96.v2.A.SL1-1	VCBP-B, VCBP	1	23,936	283
KH.C2.934.v1.A.SL1-1	Cubilin		23,900	343
KH.C9.512.v3.A.SL1-1	Villin		22,432	329
KH.C14.52.v1.A.nonSL3-1	Ci-eEF1A4, transcription elongation factor		21,904	328
KH.C1.321.v1.A.ND1-1	Cubilin		21,597	304
KH.C3.903.v1.A.SL1-1	Aldehyde dehydrogenase		20,854	306
KH.C8.575.v1.A.SL1-1	Metalloprotease		20,052	277
KH.C6.206.v1.A.ND2-1	Ci-FABP2, fatty acid-binding protein		19,509	268
KH.C5.45.v2.A.ND3-1	Glutamate dehydrogenase		19,326	281
KH.C1.742.v1.A.ND1-1	MACPF protein		18,993	264
KH.C12.207.v2.A.ND2-2	Carboxypeptidase		18,821	279
KH.C13.147.v1.A.ND1-1	Aminopeptidase N		16,364	234
KH.C1.1105.v1.A.ND1-1	Cathepsin D		14,665	196
KH.S425.11.v1.A.ND1-1	Chymotrypsin-like protease		13,863	183
KH.C7.153.v1.A.ND1-1	Ci-FABP6, fatty acid-binding protein		13,729	221
KH.S749.1.v2.A.SL1-1	Glial fibrillary acidic protein		13,721	175
KH.C9.291.v1.A.ND1-1	Ci-CLCA2, Ca-activated chloride channel	2	13,303	183
KH.C9.821.v1.A.ND1-1	Dipeptidyl peptidase		13,301	196
KH.S1458.2.v1.A.ND1-1	Serine protease		13,115	221
KH.S425.12.v1.A.ND1-1	Chymotrypsinogen		13,098	186
KH.L97.10.v1.A.nonSL1-1	Serine protease		12,890	186
KH.C3.795.v1.A.ND1-1	MACPF protein		12,851	187
KH.S425.3.v1.A.SL1-1	Chymotrypsinogen		12,485	174
KH.L6.4.v1.A.ND1-1	Peroxisomal multifunctional enzyme		12,106	162
KH.C8.679.v1.A.ND1-1	Ci-contactin, contactin		11,665	160
KH.C2.1070.v1.A.SL1-1	Tolloid-like protein		11,365	173
KH.L170.27.v2.A.ND1-1	Maltase-glucoamylase		11,014	168
KH.C12.644.v2.A.ND2-1	Glutamyl aminopeptidase		10,951	162
KH.C1.830.v1.A.ND1-1	MACPF protein		10,605	159
KH.C1.926.v1.A.ND1-1	Hemicentin	3	10,477	163
KH.C10.93.v1.A.ND1-1	Natrium/Hydrogen exchange regulatory cofactor		10,260	132
KH.C12.92.v2.A.ND1-1	Trans-1,2-dihydrobenzene-1,2-diol dehydrogenase		10,094	143
KH.C7.83.v1.A.SL3-1	Brevican core protein		8,970	159
KH.C5.460.v1.A.SL1-1	Brevican core protein		8,881	137
KH.C2.187.v3.A.SL2-2	3'-phosphoadenosine 5'-phosphosulfate synthetase		8,590	132
KH.C7.387.v1.A.ND1-1	Carboxypeptidase		8,537	127

Supplementary Table 1. Protein components of the surface matrix of chitinous barrier membrane of *C. intestinalis* Type A. This table summarizes the results of LC-MS-MS proteomic analysis of *Ciona* barrier membranes. The list is sorted with normalized protein scores. Details of the top three proteins are shown in Figure 3 and Supplementary Figure 5. This list contains 4 MACPFs and 2 VCBPs.

Bacterial division	Order	Genus	Aquarium water		Food		Stomach		Hepatic loop		Distal major coil	
			Count	(%)	Count	(%)	Count	(%)	Count	(%)	Count	(%)
**Gammaproteobacteria**			**122**	**100.0**	**472**	**93.5**	**464**	**90.1**	**316**	**79.8**	**108**	**20.5**
	Pseudomonadaceae	*Pseudomonas*	116	95.1	433	85.7	430	83.5	286	72.2	91	17.3
	Enterobacteriaceae	*Serratia*	4	3.3	25	5.0	15	2.9	19	4.8		
		Yersinia	1	0.8	4	0.8						
		unknown			7	1.4	12	2.3	4	1.0	3	0.6
		Rahnella					4	0.8				
		Plesiomonas					1	0.2	7	1.8	9	1.7
		Edwardsiella									5	1.0
	Xanthomonadaceae	*Stenotrophomonas*	1	0.8	3	0.6	2	0.4				
**Alphaproteobacteria**					**26**	**5.1**	**1**	**0.2**			**3**	**0.6**
	Acetobacteraceae	*Acetobacter*			24	4.8						
	unknown				2	0.4					2	0.4
	Caulobacteraceae	*Caulobacter*					1	0.2				
	Methylobacteriaceae	*Methylobacterium*									1	0.2
**Betaproteobacteria**					**3**	**0.6**	**4**	**0.8**	**1**	**0.3**	**22**	**4.2**
	Burkholderiaceae	*Burkholderia*			3	0.6						
	Comamonadaceae	*Delftia*					*4*	0.8			1	0.2
	Chromobacteriaceae	*Aquaspirillum*									1	0.2
	unknown								1	0.3	20	3.8
**Actinobacteria**					**2**	**0.4**						
	Propionibacteriaceae	*Propionibacterium*			2	0.4						
**Sphingobacteria**					**2**	**0.4**	**2**	**0.4**				
	Sphingobacteriaceae	*Pedobacter*			2	0.4	2	0.4				
**Fusobacteria**							**32**	**6.2**	**79**	**19.9**	**325**	**61.8**
	Fusobacteriaceae	*Cetobacterium*					32	6.2	*79*	19.9	*325*	61.8
**Bacteroidetes**							**12**	**2.3**			**39**	**7.4**
	Porphyromonadaceae	unknown					9	1.7				
	unknown						3	0.6			36	6.8
	Bacteroidaceae	*Bacteroides*									3	0.6
**Verrucomicrobia**											**27**	**5.1**
	Verrucomicrobiaceae	*Akkermansia*									27	5.1
**Clostridia**											**1**	**0.2**
	Peptostreptococcaceae	*Peptoclostridium*									1	0.2
**Planctomycetes**											**1**	**0.2**
	Planctomycetaceae	*Singulisphaera*									1	0.2
**total**			**122**	**100.0**	**505**	**100.0**	**515**	**100.0**	**396**	**100.0**	**526**	**100.0**

Supplementary Table 2. Bacterial composition of the gut microbiota of *O. mossambicus*. This table summarizes the results of a 16S rRNA gene analysis at the level of bacterial divisions, orders and genera. Items are sorted by dominance following the order of aquarium water, food, stomach, anterior intestine (hepatic loop) and posterior intestine (distal major coil). Coloring correspond to Figure 6**c**: White, gammaproteobacteria; gray, alphaproteobacteria; green, betaproteobacteria; red, fusobacteria; blue, bacteroidetes; and yellow, verrucomicrobia.

Primer name	Sequence
16SrRNA_27f	agagtttgatcctggctcag
16SrRNA_1491r	ggttaccttgttacgactt
16SrRNA_907r	ccgtcaattcctttragttt
Chs_Sep02fw1	atgtatttcagtgacagaaatg
Chs_Sep02fw2	gatggatgcaaagcttacag
Chs_Sep02fw3	ctctactacctatgtgtgcc
Chs_Sep02rv3	ggcacacataggtagtagag
Chs_Sep02rv2	aagacgctgcaaaatggg
Chs_Sep02rv1	cactggttgaaaacgcgtaag
Chs-v2_5R1	tgcccgccactgcaacctgcgtatc
Chs-v2_5R2	ttccacaagcagctccaacatccggatt
Chs-v2_3R1	agtcaggcaaaagcaagg
Chs-v2_3R2	ccgaatgtcgaaacgaagtc
Chs_3Race_Apr04	atggggaccttccacaatggcaaa
Chs_5Race_Apr04	ccctggacaatcataacaacagttgcgg
Chs5R_Sep14_01	acaacagttgcgggaccaag
Chs5R_Sep14_02	ctgtaagctttgcatccatc
Chs5R_Sep14_03	ccattgtggaaggtccccatc
Chs5Utr1_T2-1_R_392-412	ccctaactccagagtcctatg
Chs5Utr2_T2-1_R_411-431	tggtgtacctggtcataagac
Chs5Utr3_Kgr05.134.3.1_313-331	agagtcctatggtgtacct
Chs5Utr4_Kgr05.134.3.1_407-426	aactttagggtttgctctgt
Chs3Utr1_T2-2_gwR_326-345	acgcataaatgacacctacc
Chs3Utr2_T2-2_gwR_344-364	cctacattgatgtatgggcgt
Chs3Utr3_T2-2_gwR_435-455	aagtgcgagtggtaagcaaag
Macpfpf45_5Utr_f1	gatattttgtgcattgtgttgcctatagtgtac
Macpf45_5Utr_f2	cctatagtgtacaaaaatgaagattcacacaac
Macpf45_Atg_f1	atgaagattcacacaacagtattactgctg
Macpf45_Stp_r1	ttatgcatcacttctgtcacactggcttcc
Macpf45_Stp_r2	agcagttgtaatctccatttggcatctt
Macpf45_3Utr_r1	gcatacataggatcatgtaattataaacaattagttc

Macpf45_3Utr_r2	gcgaaatttcataaaagcatacataggatcatg
Vcbp_ifv-pCold_f	gtaatctctgcttaaaagcacagaatctaagatccctgccatttg
Vcbp_ifv-pCold_r	accgccacgaccttcaatgtgatggtgatggtgatgcactttatg
Vcbp_ifi_22_f	gaaggtcgtggcggtatcgtggaacaggaagtggatgttaccctg
Vcbp_ifi_349_r	ttaagcagagattacttaattgtttttcagggtaccacacggtgccg
Vcbp_ifi_278_f	gaaggtcgtggcggtgaaatcatcagtgaaaacgatcgtaac
Vcbp_ifi_280_r	ttaagcagagattacttagatgatttcaaccaccgtagattttgcatcgg
GfmM47_f1	atgaagactcagtttgtcttcatgggatgc
GfmM47_f2	gcttctatcacgcaaggtatcagtgaatcac
GfmM47_f3	cactactgagtattcagcatcaatcacaaagtc
GfmG38_f1	atggttcggtttacaagattaactgtttatg
GfmG38_f2	ccctacccattcatcaaacgtatgcagttac
GfmG37_f1	atgttggcagcccgtgcgcaaatatttgtctttg
GfmG37_f2	ggagttgtacaaagtcagcgacaatcgtc
GfmG37_r1	ttagttgatcaaatcattcatctctttggccaaatc
GfmG37_r2	cgcacaacgctctgtaagtcttcggtgtcatc
GfmG37_r3	cggtgtcatcgaagttaagcaaagtttgcagctgatg
GfmG37_rCk1	ttgtgggggttggttttactggtacagtggttg
GfmG37_rCk2	aggtagcggtacacccacaagataaaacatcg
GfmG37_rCk3	tcattgaaacaactctggtagccgtggaag
Gfm5Utr1f	caatgtcacacgcaaatgtaactgc
Gfm5Utr2f	atcagtgtgcaaaattatacgaagc
Gfm5REx1f	atgaagactcagtttgtcttcatgg
Gfm5REx2f	caaaatgaacaacggaacaagcg
Gfm5REx3f	cctgactcactactgagtattcagcatc
Gfm5REx4f	tcgaatgcacagagagctgtgg
Gfm5REx5f	aatggaggttcgtgctcagc
Gfm5R-100r	cgtttgatgaatgggtaggagcag
Gfm3Race100f	ccctgcactacatgtacgtgcc
Gfm3Race250f	ccatcttgcggagtttgtcaaaac
Gfm3Race300f	gaacacctcgtttcaaccgacg
Gfm3Race400f	gtacattcccgtccaaaccaatc
Gfm3Race_Tm65	aagcagtggtatcaacgcagag

Gfm3RaceNst_Tm65	agcagtggtatcaacgcagagtac
Gfm3Race dT30	aagcagtggtatcaacgcagagtacttttttttttttttttttttttttttttttn
GfmCk3Race_1f	cgtctgttgtgccgatggaatgg
GfmCk3Race_2f	atgccctccattcgattctgatgc
Gfm3Race_Tm56r	aagcagtggtatcaacgcagag
Gfm3RaceNst_Tm58r	aagcagtggtatcaacgcagagtac
GfmEx322-323_f1	gtgtgtgtgtcaatcagacgtcaccaaccccgatactgc
GfmEx322-323_f2	gtcaatcagacgtcaccaaccccgatactgcatgtcatg
GfmGrail405lastEx_r4	gttgttaggggttggttttactggtacagtggttgtggttaccggtg
GfmCkx_3i_r3	ccagtttcttcagttgtaggggttggttttactggtacagtggttgtgg
GfmEx3toCk_r2	ggtacagtggtctcaccagaaccagtttcttcagttgttaggggttgg
GfmEx3toCk_r1	ccagaaccagtttcttcagttgtgggggttggttttactggtacagtgg
OmChs1_f1	gattccaaacagaggtattcatttgctgtattcatcatg
OmChs1_f2	aacagaggtattcatttgctgtattcatcatggagg
OmChs1_r2	gatatcttaaagtgctatacagtcacagcggatgttta
OmChs1_r1	cttaaagtgctatacagtcacagcggatgtttatactac
OmChs1_Rt_f	tccttgtgctggtggttat
OmChs1_Rt_r	agttatctcgctgtagtctgaat

Supplementary Table 3. List of primers

Supplementary References

1. Riisgård, H. U. & Larsen, P. S. Particle capture mechanisms in suspension-feeding invertebrates. *Mar. Ecol. Prog. Ser.* **418,** 255–293 (2010).
2. Simakov et al. Hemichordate genomes and deuterostome origins. *Nature* **527,** 459–465 (2015).
3. Benito, J. & Pardos, F. in *Microscopic Anatomy of Invertebrates. Volume 15. Hemicordata, Chaetognatha, and the Invertebrate Chordates* (eds Harrison, F. W. & Ruppert, E. E.) 15–101 (Wiley-Liss, New York, 1997).
4. Bone, Q., Carré, C. & Chang, P. Tunicate feeding filters. *J. Mar. Biol. Assoc. U. K.* **83,** 907–919 (2003).
5. Petersen, J. K. Ascidian suspension feeding. *J. Exp. Mar. Biol. Ecol.* **342,** 127–137 (2007).
6. Mallatt, J. The suspension feeding mechanism of the larval lamprey *Petromyzon marinus*. *J. Zool.* **194,** 103–142 (1981).
7. Martini, F. H. in *The Biology of Hagfishes* (eds Jørgensen, J. M., Lomholt, J. P., Weber, R. E. & Malte, H.) 57–77 (Chapman & Hall, London, 1998).
8. Yalden, D. W. Feeding mechanisms as evidence for cyclostome monophyly. *Zool. J. Linn. Soc.* **84,** 291–300 (1985).
9. Goudemand, N., Orchard, M. J., Urdy, S., Bucher, H. & Tafforeau, P. Synchrotron-aided reconstruction of the conodont feeding apparatus and implications for the mouth of the first vertebrates. *Proc. Natl. Acad. Sci. U.S.A.* **108,** 8720–8724 (2011).
10. Stevens, C. E. & Hume, I. D. *Comparative Physiology of the Vertebrate Digestive System* (Cambridge University Press, Cambridge, 1995).
11. Holley, M. C. Cell shape, spatial patterns of cilia, and mucus-net construction in the ascidian endostyle. *Tissue Cell* **18,** 667–684 (1986).
12. Nielsen, S. E., Bone, Q., Bond, P. & Harper, G. On particle filtration by amphioxus (*Branchiostoma lanceolatum*). *J. Mar. Biol. Assoc. U. K.* **87,** 983–989 (2007).
13. Oku, T. & Ishikawa, K. Analysis of the hyperthermophilic chitinase from *Pyrococcus furiosus*: Activity toward crystalline chitin. *Biosci. Biotechnol. Biochem.* **70,** 1696–1701 (2006).
14. Rosado, C. J. *et al.* The MACPF/CDC family of pore-forming toxins. *Cell. Microbiol.* **10,** 1765–1774 (2008).
15. Tan, K. *et al.* Crystal structure of the TSP-1 type 1 repeats: a novel layered fold and its biological implication. *J. Cell Biol.* **159,** 373–382 (2002).
