# Supplementary material for: Chitin-based barrier immunity and its loss predated mucus-colonization by indigenous gut microbiota
Source: Nat Commun. 2018 Aug 24;9:3402. doi: 10.1038/s41467-018-05884-0 (PMC6109156; doi:10.1038/s41467-018-05884-0)
Supplement: Supplementary file 3 — Description of Additional Supplementary Files [file 41467_2018_5884_MOESM3_ESM.pdf]

Description of Additional Supplementary Files

File Name: Supplementary Movie 1
Description: **Internal filter-feeding using mucus nets by the tunicate, *Ciona intestinalis* Type A.** Water flow, generated by coordinated movements of gill slit cilia, is visualized by movements of red polystyrene beads. Beads entering the oral siphon do not exit from the atrial siphon, because they are trapped by two sheets of mucus net that are secreted from the endostyle and cover the inner walls of the pharyngeal basket. Although mucus nets are not visible due to their transparency, trapped beads help confirm the ventral-to-dorsal movement of mucus nets. When mucus nets reach the dorsal lamina, they become rolled into a mucus cord. The mucus cord is then conveyed posteriorly to enter into the esophagus and the stomach, where the cord is temporarily stored. For detailed anatomy, see Supplementary Fig. 2a–c.

File Name: Supplementary Movie 2
Description: **Internal filter-feeding using mucus nets and rotation of digesta of the lancelet, *Branchiostoma floridae*.** Water flow is visualized with movements of red polystyrene beads. Unlike filtering sheets of tunicates, mucus nets of lancelets may function as sticky attachments. In many cases, mucus nets are detached from gill bars on the way to the dorsal floor of the pharynx and reach the stomach. In the stomach, vigorous flagellate actions cause a mixing of mucus nets, trapped matter and digestive enzymes secreted from the hepatic cecum. Once entering the ilio-colon, mixed digesta start to rotate along the longitudinal axis as a mass. This rotation continues until defecation. For detailed anatomy, see Supplementary Fig. 2e.
